# Advancing subclinical keratoconus detection using polarization-sensitive optical coherence tomography and artificial intelligence

**DOI:** 10.1117/1.BIOS.3.1.015004

**Published:** 2026-02-09

**Authors:** Rahul P. Patil, Raghav Narasimhan, Anchana Pisharody, Rohit Shetty, Pooja Khamar, Vinodhini Devendiran, Rudy M.M.A. Nuijts, Abhijit Sinha Roy

**Affiliations:** a Narayana Nethralaya Foundation, Imaging, Biomechanics and Mathematical Modelling Solutions Laboratory, Bangalore, Karnataka, India; b Maastricht University Medical Center, Department of Ophthalmology, Maastricht, The Netherlands; c Narayana Nethralaya Eye Hospital, Department of Cornea and Refractive Surgery, Bangalore, Karnataka, India

**Keywords:** ps-oct, polarization-sensitive, cornea, artificial-intelligence, keratoconus, classification.

## Abstract

**Significance:**

Early detection of keratoconus (KC), a progressive corneal disorder, remains a major clinical challenge. Polarization-sensitive optical coherence tomography (PS-OCT) is an advanced imaging technique that can quantify corneal birefringence, offering insight into collagen organization and microstructural integrity.

**Approach:**

A total of 359 eyes were examined and evaluated. This study explores PS-OCT-derived phase retardation (PR) and corneal sublayer thickness as potential diagnostic biomarkers for healthy, subclinical (SKC), and KC eyes. Further, the performance of AI-based classification models developed from PS-OCT, Pentacam, and MS-39 data were compared, using random forest classifier trained with a leave-one-out methodology and identical hyperparameter settings.

**Results:**

All AI models demonstrated comparable accuracy among devices for healthy and KC detection. However, SKC classification differed from Pentacam and MS-39. Here, 39.5% of the SKC eyes were reclassified as healthy by PS-OCT, compared with 27.5% by Pentacam and 30.3% by MS-39. The average diagnostic performance of PS-OCT included an AUC, precision, recall, F1 score, and accuracy of 0.91, 83%, 82%, 0.82, and 82%, respectively. For the Pentacam, the same were 0.95, 87%, 86%, 0.86, and 86%, respectively. For the MS-39, the same were 0.94, 86%, 86%, 0.85, and 86%, respectively.

**Conclusions:**

Overall, AI model agreement was strong for healthy and KC groups but varied in SKC. PS-OCT provides complementary diagnostic value and may refine subclinical KC detection for safer refractive surgery decisions.

Statement of Discovery This work shows a large sample size clinical application of ultrahigh-resolution polarization sensitive OCT for advanced diagnoses of keratoconus corneas using artificial intelligence. This could advance diagnoses of sub-clinical disease with corneal birefringence, which are challenging to diagnose with routine tomography only.

## 1 Introduction

Polarization-sensitive optical coherence tomography (PS-OCT) measures tissue birefringence by detecting differences in refractive index for polarized light aligned parallel or orthogonal to collagen fibers.^1^^,^^2^ Birefringence results from the anisotropic structure of collagen lamellae, which behave as birefringent films with their slow axis aligned to the collagen fiber direction.^3^^,^^4^ Early studies using electron microscopy and X-ray scattering established that corneal collagen fibers in the posterior human stroma are arranged orthogonally in the central cornea and adopt a circumferential pattern near the limbus.^3^^,^^5^^,^^6^ More recently, Li et al. elegantly quantified local lamellar orientation and stromal microstructure in sheep corneas *ex vivo* using PS-OCT.^7^ This organized structure leads to low retardance and a preferred slow axis near the corneal apex, as incident light is nearly normal to the central cornea.^3^ Toward the periphery, the circumferential lamellae orientations prevent compensation of birefringence between successive layers, increasing retardance.^3^ PS-OCT allows *in vivo* quantification of these depth-dependent birefringence properties by measuring retardance per unit depth.^1^ In keratoconus (KC), the preferred fiber orientation notably changes in the cone region, resulting in increased birefringence in the central cornea.^8^^,^^9^

A recent study showed that higher birefringence was related to more aligned fibers and larger collagen fiber diameter.^10^ A larger collagen fiber diameter indicates a higher collagen content and thicker collagen lamellae.^10^ Disruption in the collagen fiber mesh is the primary and possibly early cause of structural failure in KC, leading to thinning and curvature steepening.^8^^,^^11^ Therefore, imaging of this collagen fiber mesh via direct or indirect indices, such as corneal birefringence or phase retardation (PR), can provide vital information about early KC.^9^^,^^12^^,^^13^ Intracorneal layers can also undergo thinning in addition to stromal thinning. In addition to remodeling of the epithelium, both the Bowman’s layer (BL) and Descemet’s membrane (DM) also undergo thinning in early KC.^9^^,^^14^^,^^15^ Thinning is a notable feature because tissue thickness can influence biomechanical stiffness, which is a measure of the tissue’s resistance to deformation under load. However, stiffness also depends on the intrinsic material properties of the corneal layers, which may change during disease progression.^16^ Thus, polarization-sensitive measurements and intra-corneal layer thickness could provide important biomechanical information relevant to disease progression.^9^

Building on our preliminary pilot studies, this study expands to a larger dataset that includes healthy, subclinical, and KC corneas, all of which are examined using a custom-built ultrahigh-resolution polarization-sensitive OCT (PS-OCT).^9^^,^^17^ We measured and constructed the spatial distribution of PR derived from the corneal stroma. Further, we measured the spatial distribution of epithelial and BL thicknesses in all the corneas. Custom indices were defined and analyzed using a random forest (PS-RF) artificial intelligence model. All patients also underwent corneal tomography assessment using the Pentacam HR (OCULUS Optikgeräte GmbH, Germany) and MS-39 (CSO Italia, Italy) devices. The Pentacam HR employs a rotating Scheimpflug camera to measure corneal curvature, elevation, thickness, refractive power, and lens density in the anterior segment of the eye. In contrast, the MS-39 is a hybrid tomographer that combines Placido disc technology, which uses patterned ring illumination reflected off the cornea to map anterior surface topography, with spectral domain OCT to provide detailed evaluations of corneal pachymetry, elevation, epithelial thickness, anterior chamber depth, and aberrations. Indices from Pentacam HR and MS-39 devices were also analyzed using a random forest (PC-RF and MS-RF, respectively) artificial intelligence model. This facilitated direct comparisons between the diagnostic accuracy of devices and the assessment of potential reclassification (if any) of eyes.

## 2 Methods

### 2.1 Overview

This retrospective study analyzed medical records of patients who visited the outpatient department of Narayana Nethralaya Eye Hospital between January 2022 and September 2024. The study was approved by the Narayana Nethralaya Ethics Committee, Bangalore, India, and adhered to the principles outlined in the Declaration of Helsinki. All subjects in this study underwent comprehensive ophthalmologic evaluations, including refractive error, slit-lamp examinations, and imaging using three devices: Pentacam (OCULUS Optikgeräte GmbH), MS-39 (CSO, Italy), and a custom-built ultrahigh resolution and polarization sensitive optical coherence tomography (PS-OCT) device.

### 2.2 PS-OCT Device and Data Processing

The PS-OCT device is a custom-built spectral domain OCT using a center wavelength of 840 nm and a bandwidth of 100 nm.^9^^,^^18^ In tissue, the axial resolution was 2.96  μm and the depth measurement range was 2.9 mm, assuming a refractive index of 1.385.^9^^,^^18^ The sensitivity was 97 dB at a 50 kHz A-scan rate. The size of the corneal scans was 10×8  mm using a raster scan protocol to acquire a volume of data comprising 1024×64 A-scans (horizontal × vertical directions).^9^ The scan field of 10 mm diameter was achieved using a conical scan setup that provided a near-uniform signal strength throughout the cornea. The total incident power on the cornea was 1.2 mW.

The polarization state of light in the sample and reference arms was set using quarter-wave plates at 45 deg and 22.5 deg, respectively. This allowed circularly polarized light to illuminate the sample and provided equal reference power in the two orthogonal polarization channels. Our prior works provide further details regarding the system design and data processing methods.^9^^,^^18^ The polarization-sensitive information was derived in the form of reflectivity (R) and PR using the following equations:

$$R(z)∼A_{1}{\left(z\right)}^{2}+A_{2}{\left(z\right)}^{2},$$ (1)

$$\mathrm{PR}(z)=\arctan(\frac{A_{2}(z)}{A_{1}(z)}),$$ (2)

where $A_{1}$ and $A_{2}$ represented the amplitudes of the complex signals obtained using the Fourier transform of the spectral data from the two orthogonal polarization channels, and z was the depth coordinate. All scans were acquired by a single operator.

The PR *en face* maps were generated from the posterior surface of the cornea, as this layer is polarization preserving and enables the observation of gross polarization changes introduced by the arrangement of collagen lamellae in the stroma. Corneal layer segmentation in this study was performed using the reflectivity output. The details on the generation of layer thickness mapping can be found in our previous work.^9^^,^^19^ The PR and thickness en face maps were smoothened by applying a floating average filter equivalent to a 1×1  mm area of the cornea. The Bowman’s layer can extend beyond 9 mm in diameter before thinning and merging into the cornea. To ensure segmentation uniformity for epithelium and Bowman’s layer thickness mapping, we considered an 8×8  mm field along the x- and y-axes, whereas PR maps were extended to a field size of 10×8  mm.^9^^,^^19^

### 2.3 Classification of Healthy, SKC, and KC Eyes

Eyes in the study were categorized into three groups: healthy, subclinical keratoconus (SKC), and KC. The classification was based on criteria established by Randleman et al. utilizing diagnostic parameters derived from Pentacam data, including the KISA% index, inferior-superior (IS) value, and Belin/Ambrosio display total deviation (BAD-D) score.^20^

The healthy group comprised eyes from subjects who were clinically assessed to be in a healthy condition, with an uncorrected distance visual acuity of 20/20 and showing no signs of disease. They met the following criteria: a KISA% index of less than 60%, an IS value below 1.4, and a BAD-D score under 1.65.^20^ The SKC group comprised of fellow eyes of highly asymmetric KC subjects (n=33) and eyes from bilateral suspected KC subjects (n=76). All eyes met the criteria of a KISA% index below 60%, an IS value under 1.6, and a BAD-D score less than 2.6.^20^ The fellow eyes of highly asymmetric KC subjects showed no clinical signs of disease and no abnormalities on slit-lamp examination. Eyes from bilateral suspected KC subjects showed no obvious KC symptoms or abnormal slit-lamp findings in either eye. However, suspicious corneal imaging findings were observed, including asymmetric anterior curvature assessed using a fixed scale of 0.5 diopters (D).^20^

The KC group comprised subjects with clinically evident KC. These eyes exhibited characteristic features, such as corneal thinning visible during a slit-lamp examination. The diagnostic criteria for this group included a KISA% index exceeding 60%, an IS value greater than 3.0, and a BAD-D score above 3.0.^20^ Scans with poor quality due to artifacts such as blinking, eyelashes, or movement were excluded. In addition, participants were excluded if they had a history of prior ocular surgeries, wore contact lenses, were using topical medications, were pregnant, or had other corneal pathologies or systemic diseases affecting the cornea.

### 2.4 AI Model

In this study, we developed three AI models using datasets derived from different imaging modalities: PS-OCT, Pentacam, and MS-39. The MS-39 uses a combination of Placido and OCT for topography and tomography, respectively. Acquisitions across the three devices were performed on the same day by a single operator. The input features for the three models also varied depending on the device used.

In our previous studies, we demonstrated that Zernike polynomials up to the 6th order can effectively represent 3D spatial mapping of thickness distributions.^21^ The spatial mapping described an increase in the thickness from the center to the periphery in healthy corneas and focal thinning in KC corneas. This was achieved by expressing the spatial variation in thickness as a function of radius and meridian. The spatial profiles of the individual Zernike polynomials are presented in Fig. S1 in the Supplementary Material as the Zernike pyramid. Table 1 gives a summary of all the features used in the three device AI models. The PS-OCT AI model (Table 1) used only the spatial distribution of PR, epithelium, and Bowman’s layer thickness distribution mapped by Zernike polynomials (a total of 84 features). The Pentacam AI model (Table 1) included anterior surface keratometries, derived indices, and total corneal wavefront aberrations (Zernike polynomials of order 6). The MS-39 AI model (Table 1) included anterior surface keratometries and wavefront aberrations (Zernike polynomials of order 6), epithelium-Bowman’s interface keratometries and wavefront aberrations, and epithelium thickness distribution (Zernike polynomials of order 6). While the Pentacam and MS-39 acquired data along 25 and 12 meridians, respectively, the PS-OCT acquired data along 64 B-scans (raster scans). Since the devices used different definitions of the geometric center of the cornea, we did not use local measures such as central epithelium thickness in the AI models.

**Table 1:** Overview of features from PS-OCT, Pentacam, and MS-39 used in the AI models.

Device/model	Category	Features	n
PS-OCT	Spatial distribution of phase retardation, epithelium layer, Bowman’s layer thickness	Zernike polynomial coefficients up to order 6.	28×3
Pentacam	Anterior surface keratometry	K1, K2, K mean, K max, Axis (flat), Astigmatism.	32
	Derived indices	ISV, IVA, KI, CKI, IHA, IHD.	
	Wavefront aberrations cornea (front)	RMS HOA, RMS LOA, Astigmatism 0 deg, Defocus, Astigmatism 45 deg, Trefoil 0 deg, Coma 0 deg, Coma 90 deg, Trefoil 30 deg, Spherical Aberration.	
	Wavefront aberrations cornea (back)	RMS HOA, RMS LOA, Astigmatism 0 deg, Defocus, Astigmatism 45 deg, Trefoil 0 deg, Coma 0 deg, Coma 90 deg, Trefoil 30 deg, Spherical Aberration.	
MS-39	Anterior surface keratometry	AntK1, AntK2, AntAxis, AntKmax.	23
	Anterior surface wavefront aberrations	AntLORMS, AntHORMS, AntComaRMS, AntDefocus, AntSA.	
	Epithelium-Bowman interface keratometry	BWK1, BWK2, BWAxis, BWKmax.	
	Epithelium-Bowman’s layer wavefront aberrations	BWLORMS, BWHORMS, BWComaRMS, BWDefocus, BWSA.	
	Epithelium thickness distribution mapped by Zernike polynomials	eLORMS, eHORMS, eComaRMS, eDefocus, eSA.	

Ant – Anterior corneal surface; BW – Epithelium-Bowman’s layer interface; e – Epithelium thickness K1, K2 – steep and flat axis curvature; K mean – mean keratometry (K1, K2); K max – maximum keratometry; ISV -, IVA -, KI – Keratoconus index, CKI – Central keratoconus index, IHA – Index of height Asymmetry, IHD – Index of Height decentration, IS Value – inferior superior value; RMS – root mean square; HOA – higher order aberration; LOA – lower order aberration; LORMS – root mean square of lower order Zernike terms; HORMS – root mean square of higher order Zernike terms; SA – Primary spherical aberration (4th order); ComaRMS – root mean square of coma Zernike terms.

The distribution of features across all three groups for the specified three models is presented as Tables S1(a)–S1(c) in the Supplementary Material.

To enhance the robustness and reliability of the results, all three AI models (PS-RF, PC-RF, and MS-RF) were built using the leave-one-out cross-validation technique with identical hyperparameters. This methodology involved iteratively training the model on all but one data point and validating it on the excluded data point. Further, all RF classifiers were implemented using the default settings provided by the Python 3 scikit-learn package (version 1.5.2). The following hyperparameters were used: n_estimators = 100 (indicating the number of trees in the forest), criterion=”gini” (using the Gini impurity as the criterion for splitting nodes), max_depth = None (allowing the trees to grow until all leaves are pure or until they contain fewer than the minimum number of samples), min_samples_split = 2 (specifying the minimum number of samples required to split an internal node), and min_samples_leaf = 1 (ensuring that each leaf node has at least one sample).

### 2.5 Statistical Analyses

All parameters were summarized as mean ± standard deviation followed by the range (min, max) in square brackets after assessment of normality of distribution using the Kolmogorov-Smirnov test. The differences in parameters across groups were assessed using one-way ANOVA and independent sample t-tests. A p-value less than 0.05 was considered statistically significant. Proportions were compared with the chi-square test. MedCalc v23.0.9 (MedCalc Inc., Ostend, Belgium) software was used for statistical analyses.

For the AI models, the area under the curve (AUC), precision, recall, F1-score, and accuracy were obtained. For each eye group, statistically significant differences between the AI models were quantified by constructing the receiver operating characteristics (ROC) curve from the predicted probability scores for each eye within a group. A p-value less than 0.05 was considered statistically significant.

## 3 Results

We used 359 eyes from 197 subjects that met the inclusion criteria to train the three AI models. The cohort comprised 120 healthy eyes, 109 SKC eyes, and 130 KC eyes. Among the SKC group, 33 eyes were the fellow eyes of highly asymmetric KC subjects. Details of the demographics and clinical characteristics of the groups are provided in Table 2. Age was statistically different between the groups (p=0.002), but this difference was not clinically significant. The proportion of left and right eyes was similar between the eye groups (p>0.05). The male-to-female ratio was similar between the healthy and SKC groups (p>0.05) but was different between the SKC and KC groups (p<0.0001). The sphere and cylinder of KC eyes were different from healthy and subclinical eyes (p<0.001). Similarly, Pentacam and MS-39 parameters showed significant differences between the eye groups (p<0.001). The three AI models were trained and tested on the data from the three devices. For the PS-OCT, the average (for the three eye groups) area under the curve (AUC), precision, recall, F1-score, and accuracy were 0.91, 83%, 82%, 0.82, and 82%, respectively. For the Pentacam, the values were 0.95, 87%, 86%, 0.86, and 86%, respectively. For the MS-39, the values were 0.94, 86%, 86%, 0.85, and 86%, respectively. For the classification of healthy eyes, the three AI models achieved similar proportions (p>0.05). A similar result was obtained for the classification of KC eyes. However, the three models differed significantly when it came to the classification of SKC eyes (p<0.001). The mean and standard deviation (minmax) of each of the AI model training parameters are listed in Tables S1(a)–S1(c) in the Supplementary Material.

**Table 2:** Demographics (n=359) of the study groups.

Parameter	Healthy (n=120)	Sub-clinical (n=109)	Keratoconus (n=130)	p-value^a^
Age (years)	29 ± 6 (18 to 47)	30 ± 8 (13 to 48)	27 ± 7 (11 to 47)	0.002^b^
Eye (Right, Left)	66 (55%), 54 (45%)	51 (46%), 60 (54%)	61 (47%), 69 (53%)	—
Gender (male, female)	54 (45%), 66 (55%)	43 (39%), 68 (61%)	96 (74%), 34 (26%)	—
Sphere (D)	−3.9 ± 2.5 (−10.25 to 2.00)	−3.1 ± 2.8 (−11.00 to 7.00)	−0.8 ± 2.7 (−20.00 to 5.50)	<0.001^c^
Cylinder (D)	−1.2 ± 1.1 (−5.50 to 0.00)	−1.1 ± 1.0 (−4.50 to 0.00)	−3.0 ± 2.0 (−9.00 to 0.00)	<0.001^c^
MRSE (D)	−4.5 ± 2.5 (−11.50 to 0.62)	−3.7 ± 3.0 (−13.25 to 7.00)	−2.3 ± 2.8 (−20.00 to 4.00)	<0.001^c^
CDVA (logMAR)	0.01 ± 0.04 (0.00 to 0.30)	0.02 ± 0.06 (0.00 to 0.40)	0.13 ± 0.17 (0.00 to 1.20)	<0.001^c^
**PENTACAM**				
IS Value	0.3 ± 0.5 (−0.99 to 1.33)	0.4 ± 0.5 (−0.68 to 1.36)	5.8 ± 2.3 (3.02 to 13.43)	<0.001^c^
KISA%	8 ± 11 (0.33 to 53.33)	8 ± 10 (0.33 to 51.83)	912 ± 1290 (81.27 to 6663.67)	<0.001^c^
BAD D score	1.0 ± 0.4 (0.00 to 1.61)	1.9 ± 0.5 (0.18 to 2.57)	8.1 ± 2.8 (3.68 to 21.25)	<0.001^d^
K max (D)	44.5 ± 1.2 (42.10 to 47.80)	46.0 ± 1.9 (41.40 to 50.30)	55.0 ± 4.9 (45.50 to 72.00)	<0.001^d^
TCT (μm)	531 ± 28 (485 to 605)	519 ± 38 (420 to 594)	471 ± 40 (383 to 579)	<0.001^d^
CCT (μm)	534 ± 28 (489 to 607)	522 ± 38 (426 to 601)	490 ± 40 (403 to 597)	<0.001^d^
ART Max	432 ± 55 (308 to 583)	376 ± 73 (243 to 590)	163 ± 42 (77 to 283)	<0.001^d^
**MS-39**				
CCT (μm)	536 ± 28 (484.80 to 603.70)	524±39 (426.40 to 603.10)	490 ± 40 (407.40 to 593.70)	<0.001^c^
CET (μm)	54.9 ± 3.0 (48.20 to 62.90)	54.0 ± 3.6 (47.50 to 66.80)	49.9 ± 6.4 (27.70 to 66.10)	<0.001^c^
Kmax (D)	44.8 ± 1.2 (42.26 to 48.02)	46.4 ± 1.9 (41.83 to 50.22)	54.6 ± 4.7 (45.53 to 75.04)	<0.001^d^

Data are represented as mean ± standard deviation, and range (min–max) appear in parentheses.

a One-Way ANOVA.

b KC versus Sub-clinical.

c KC versus Healthy and Sub-clinical.

d KC versus healthy versus sub-clinical; CDVA = Corrected distance visual acuity; D = diopter; logMAR = logarithm of the minimum angle of resolution; Kmax = maximum keratometry; MRSE = manifest refraction spherical equivalent; TCT = thinnest corneal thickness; ART = Ambrosio relational thickness.

**Fig. 1:**
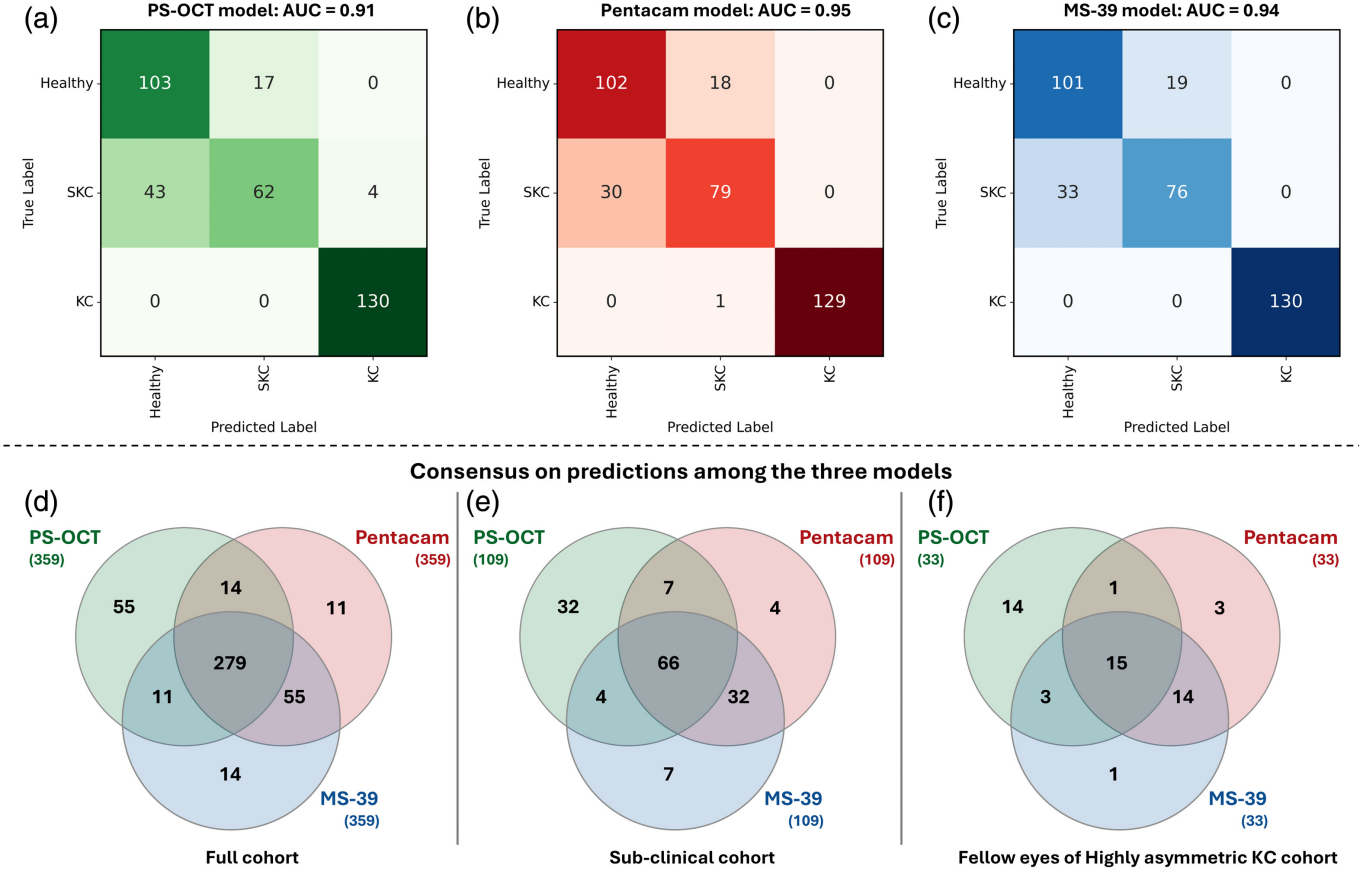
Model performance and classification outcomes.

We constructed confusion matrices [Figs. 1(a)–1(c)] and Venn diagrams [Figs. 1(d)–1(f)] to evaluate differences between the models. For the healthy eyes, the performance of all three models was nearly identical, with PS-OCT, Pentacam, and MS-39 achieving accuracies of 85.8% (103/120), 85% (102/120), and 84.2% (101/120), respectively. Similarly, for KC eyes, the models performed almost uniformly, with PS-OCT and MS-39 both reaching 100% accuracy (130/130) and Pentacam attaining 99.2% (129/130). In contrast, the SKC group revealed significant variability among models. The accuracy for PS-OCT was 56.9% (62/109), significantly lower than Pentacam, which achieved 72.5% (79/109, p=0.029). The MS-39 model showed intermediate performance with 69.7% accuracy (76/109). The Venn diagrams offer a visual representation of agreement between the AI models. For the entire cohort [Fig. 1(d)], all three models predicted the same class in 279 out of 359 eyes (77.7%). Pairwise concordance identified 55 eyes classified identically by MS-39 and Pentacam, 11 by MS-39 and PS-OCT, and 14 by PS-OCT and Pentacam. For SKC eyes specifically [Fig. 1(e)], the three models agreed on 66 out of 109 cases (60.6%). For the fellow eyes of asymmetric KC patients [Fig. 1(f)], agreement declined further, with only 15 out of 33 cases (45.5%) classified identically across all models. A recurring pattern in both Figs. 1(e) and 1(f) was the discordance between PS-OCT and the other two systems. Pentacam and MS-39 consistently showed higher agreement, classifying 32 SKC eyes and 14 fellow eyes identically. By comparison, both PS-OCT and Pentacam, as well as PS-OCT and MS-39, agreed on fewer than 10 eyes in these subgroups. It is important to note that the dataset in Fig. 1(f) is a direct subset of the eyes represented in Fig. 1(e).

**Fig. 2:**
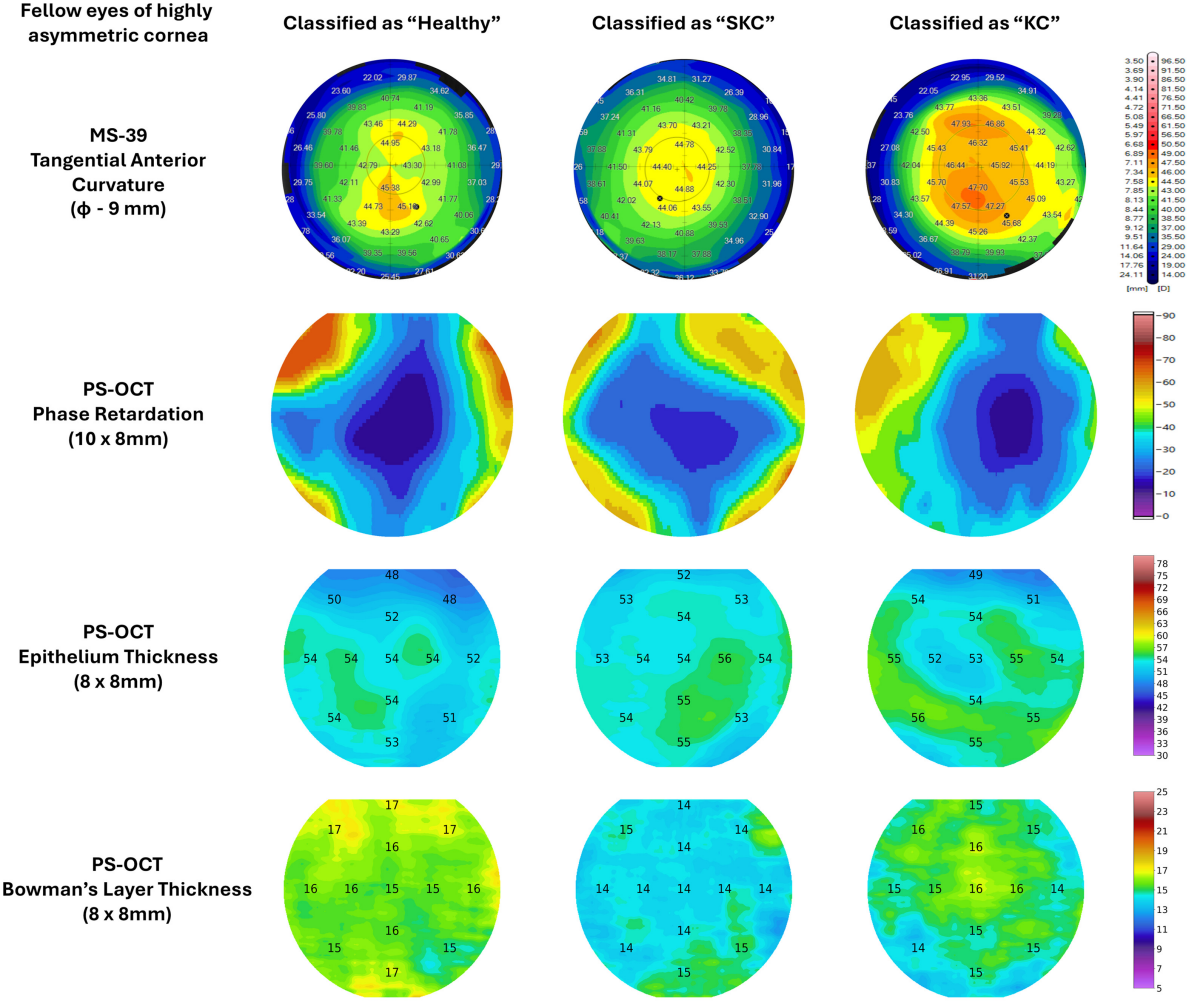
Representative examples of SKC eyes classified as healthy, SKC and KC.

Table 3 shows the mean and standard deviation (min-max) of some of the parameters from the three devices for the SKC eyes classified as healthy and *“true”* SKC by the PS-OCT AI model. Despite the SKC eyes classified as SKC having greater stromal and corneal thickness (column 2 of Table 3), these eyes had greater PR, BAD-D, IS value, and Kmax (anterior surface and epithelium-Bowman’s interface) than the SKC eyes classified as healthy (p<0.05). Figure 2 illustrates these trends in the fellow eyes of asymmetric KC patients. The 1st column of Fig. 2 shows a fellow eye classified as healthy. The 2nd and 3rd columns of Fig. 2 show a fellow eye classified as SKC and KC, respectively. The classification was assigned by the PS-OCT AI model. The healthy eye had a rhombus-shaped PR map, almost uniform epithelium thickness, and a very healthy Bowman’s layer thickness (15 to 17  μm). The SKC eye also had a rhombus-shaped PR map, but the PR magnitudes in the central cornea were greater than the PR magnitudes in the healthy eye, e.g., the area of the dark blue zone was greater in the healthy eye (Fig. 2). The SKC eye also had uniform epithelium thickness and a significant but uniform reduction in Bowman’s layer thickness (14  μm) relative to the healthy eye. The tangential curvature of both healthy and SKC eyes was uncharacteristic of KC eyes (Fig. 2). The eye classified as KC [3rd column in Fig. 2] had a highly distorted PR map, a doughnut-shaped epithelium thickness map, and a highly nonuniform Bowman’s layer thickness map.

**Table 3:** Parameters of subclinical keratoconus eyes reclassified as healthy and “true” subclinical by the PS-OCT AI model.

Parameter	Healthy (n=43)	Sub-clinical (n=62)	P-value^a^
**PS-OCT**			
PR (degrees)	27.8 ± 3.4 (20.19 to 34.51)	31.6 ± 3.7 (24.97 to 41.65)	0.001^b^
ELT (μm)	52.3 ± 3.0 (47.48 to 60.67)	52.0 ± 3.5 (44.64 to 59.82)	0.566
BLT μm)	15.8 ± 1.8 (12.39 to 20.50)	15.7 ± 1.6 (13.15 to 19.28)	0.978
SLT (μm)	458 ± 38 (366.57 to 523.00)	477 ± 39 (367.95 to 562.03)	0.013^b^
CT (μm)	526 ± 40 (433.97 to 603.04)	545 ± 40 (437.59 to 631.54)	0.019^b^
**Pentacam**			
BAD-D	1.7 ± 0.7 (0.18 to 2.56)	2.0 ± 0.4 (1.03 to 2.57)	0.025^b^
IS Value	0.6 ± 0.5 (−0.42 to 1.36)	0.3 ± 0.5 (−0.68 to 1.30)	0.008^b^
KISA	10 ± 12 (0.33 to 51.83)	7 ± 9 (0.42 to 42.46)	0.132
Kmax (D)	45.1 ± 1.7 (41.40 to 48.10)	46.6 ± 1.9 (42.30 to 50.30)	0.001^b^
TCT (μm)	510 ± 39 (421.00 to 584.00)	525 ± 37 (420.00 to 594.00)	0.062
CCT (μm)	515 ± 39 (428.00 to 589.00)	527 ± 37 (426.00 to 601.00)	0.097
ISV	20 ± 4 (11.00 to 30.00)	21 ± 7 (9.00 to 39.00)	0.136
IVA	0.15 ± 0.04 (0.06 to 0.26)	0.13 ± 0.07 (0.02 to 0.35)	0.067
IHA	7 ± 5 (0.00 to 19.00)	6 ± 5 (0.20 to 21.00)	0.217
IHD	0.01 ± 0.01 (0.00 to 0.02)	0.01 ± 0.01 (0.00 to 0.03)	0.344
**MS-39**			
AntKmax (D)	45.4 ± 1.7 (41.83 to 48.34)	47.0 ± 1.9 (42.95 to 50.22)	0.001^b^
BWKmax (D)	46.0 ± 1.6 (42.01 to 48.93)	47.6 ± 1.8 (43.13 to 51.27)	0.001^b^
CET (μm)	53.8 ± 3.6(47.50 to 62.20)	54.2 ± 3.8 (47.50 to 66.80)	0.583
CCT (μm)	516 ± 39 (428.50 to 595.30)	529 ± 38 (426.40 to 603.10)	0.098

PR = Phase retardation; ELT = Epithelium layer thickness; BLT = Bowman’s layer thickness; SLT = Stromal layer thickness; CT = Corneal thickness; CET = Central epithelium thickness; CCT = Central corneal thickness; μm = micro-meter; Data of PR, ELT, BLT, SLT, CT in this table is derived from zone up to 6 mm.

Data are represented as mean ± standard deviation, and range (min–max) appear in parentheses.

a Independent sample t-test.

b indicates significant difference between groups.

SKC subgroup analysis reveals PS-OCT’s bidirectional discrimination across fellow eyes and bilateral suspects from the SKC cohort (n=109) [see Tables S2(a)–S2(b) in the Supplementary Material]. In 33 fellow eyes from highly asymmetric KC cases, Pentacam and MS-39 classified 12/33(36%) and 16/33(48%) as healthy, respectively, whereas PS-OCT identified 21/33(64%) as healthy. In addition, PS-OCT detected two subtle KC cases (FHA#16,33) that both MS-39 and Pentacam models classified as SKC. The Pentacam and MS-39 classified 18/76(24%) and 19/76(25%) as healthy in 76 bilateral suspects, in contrast to PS-OCT’s more conservative 22/76(29%). PS-OCT uniquely identified 2 additional KC cases (BI#33, 66) completely missed by topography-driven models, where Pentacam and MS-39 exhibited uncertainty between healthy and SKC classifications.

## 4 Discussion

Advances in corneal imaging have significantly improved the way we understand and diagnose KC, a complex and multifactorial disease. Earlier, clinical practice relied mainly on corneal topography, but with newer technologies, the field has moved toward hybrid approaches combining topography and tomography, which improved sensitivity for detecting early disease. However, indices based on shape alone cannot capture the subtle biomechanical or microstructural changes that occur before visible corneal deformation appears.^22^ Tomography remains central for measuring global corneal geometry, but its limitations create a diagnostic gap, especially for early detection. PS-OCT may help address this issue by combining ultrahigh-resolution tomography with polarization-sensitive functional imaging, thereby contributing to a more frank assessment of true SKC. This allows both structural and microstructural information to be obtained simultaneously and provides complementary diagnostic value that can potentially enable earlier and more reliable detection. Therefore, in this study, we evaluated PS-OCT along with established devices for KC classification, focusing particularly on the subclinical range. Two important considerations arise when interpreting our results. First, the target classification of healthy, subclinical, and KC remains difficult, as the literature contains multiple criteria and often conflicting definitions. This inconsistency is important because any AI-based diagnostic tool is limited by the quality of the definitions it is trained on. We attempt to bridge this concern by using a more recent classification methodology provided by Randleman et al., which uses a combination of multiple indices for defining the classes instead of one single parameter.^20^

There has been increasing interest in imaging corneal sub-layers to improve SKC detection. Studies on unilateral KC patients showed that epithelial thickness indices alone performed poorly (AUC=0.68)^23^ and even when combined with tomography in AI models, sensitivity remained limited (71.5%).^21^ By contrast, Bowman’s layer may be more sensitive, as even a small thinning of 1  μm (from an average baseline of ≈15  μm) represents a significant percentage reduction (6.6%) in healthy corneas.^9^ Detecting such changes requires ultrahigh-resolution imaging, which not only allows observation of small structural changes but also improves repeatability of measurements.^19^ Several studies have confirmed the diagnostic value of Bowman’s layer thickness in SKC and KC.^9^^,^^14^^,^^24^^,^^25^ However, Pircher et al. reported that Bowman’s layer may show thickening in unilateral KC,^25^ although we did not observe this in our present or past work.^9^^,^^19^ A thicker Bowman’s layer could also act as a physical barrier, limiting inflammatory signals from the epithelium and slowing stromal degeneration. Shi et al. showed that incorporation of the thickness of the epithelium, Bowman’s layer, and stroma performed better than Pentacam indices in AI models.^24^ Based on this evidence, we included the epithelial and Bowman’s layers as key features in the PS-OCT AI model. Previous studies also suggest that focal thinning of the epithelium, Bowman’s layer, or both serves as a stronger SKC marker than global thickness, improving discrimination from healthy corneas and further refining refractive surgery eligibility criteria.^9^^,^^14^^,^^26^

The second consideration is that direct comparisons across devices remain difficult because parameters are not standardized. To address this, AI models were built separately for each device using identical leave-one-out methodology and hyperparameter configuration. In PS-OCT, PR, the epithelial, and Bowman’s layer thicknesses were spatially mapped and analyzed through Zernike polynomials. Although relatively simple, these descriptors effectively represented corneal spatial variations. To our knowledge, this is the first study to assess PR and Bowman’s layer spatial features in this systematic manner. Corneal birefringence, derived from PR, is a novel marker reflecting stromal collagen organization. Corneal thickness has traditionally been seen as a risk factor, but many thin corneas remain stable and display normal topography.^9^ Our earlier work confirmed that such corneas may also maintain normal PR as well as normal epithelial and Bowman’s thickness profiles, showing that reduced thickness alone does not always imply ectasia risk. In the current study, the ground truth for identifying SKC, as outlined in the methods section, was established using indices that are well-validated and routinely implemented in clinical practice. Both the Pentacam and MS-39 AI models rely on comparable tomographic features, and classified 30 and 33 SKC eyes, respectively, as healthy. Since the ground truth for SKC classification is based primarily on Pentacam tomographic indices, these numbers reflect the inherent limitations of this baseline diagnostic standard. The PS-OCT AI model classified 43 SKC eyes as healthy, potentially true unilateral cases. Thus, advanced polarization-sensitive imaging and sublayer features yielded a 33% relative improvement over conventional tomography in identifying true unilateral KC patients. This was also supported by the SKC subgroup analysis that demonstrated PS-OCT’s bidirectional discrimination across fellow eyes and bilateral suspects from the SKC cohort (n=109) [see Tables S2(a)–S2(b) in the Supplementary Material] by reclassifying SKC to Healthy where appropriate and also detecting KC eyes that were completely missed by conventional methodologies. This suggests that PS-OCT may identify subtle structural differences that refine or challenge the existing ground truth, which was established with conventional tomography. However, further longitudinal studies remain essential to validate these reclassifications against actual progression outcomes. Beyond the stratification criteria used in this study, eyes with naturally thin corneas are routinely classified as suspects, despite normal anterior and posterior topographies, epithelial, and Bowman’s layer thickness profiles. Further, our prior study using the PS-OCT challenged the notion of classifying corneas as suspects based solely on low thickness.^9^ These observations demonstrate that PS-OCT provides complementary diagnostic insights, potentially enabling a more comprehensive characterization of corneal microstructural changes underlying subclinical disease. Further, PR offers a quantitative measure of the organization of collagen fibers and lamellar structures, both of which have been shown to play a critical role in determining the biomechanical strength of the cornea.^10^^,^^27^

Previous attempts with biomechanics or Brillouin imaging also highlight the ongoing search for better early markers of KC. The combination of Pentacam and Corvis-ST has contributed to the development of tomographic and biomechanical indices such as the TBI and CBI.^28^ Studies consistently report that TBI performs better than CBI, although AUC values for TBI vary widely (0.65 to 0.99), highlighting the overlap between SKC and healthy populations and consistent with our findings (Fig. 1 and Table 2). Brillouin imaging has also shown potential, with some reports of perfect diagnostic discrimination (AUC 1.0).^20^ However, further examination of the supplementary data used in the study revealed a strong negative correlation between Brillouin shifts and central corneal thickness in both the central 2 mm and central 5 mm zones, across a total sample size of 30, suggesting that thickness may be a confounding factor.^20^ In a recent study on Brillouin shift, CCT and TCT were significantly different between healthy, SKC, and KC eyes (p<0.001).^28^ Likewise, most Brillouin parameters were significantly different between the groups (p<0.001) and decreased as the thicknesses also decreased from healthy to KC eyes p<0.001).^28^ Another concern raised by peer groups relates to the influence of water content on Brillouin imaging.^29^^,^^30^ A comparative study evaluating corneal hydration effects across Brillouin spectroscopy, Raman spectroscopy, and PS-OCT reported that PS-OCT measurements were independent of corneal hydration, in contrast to Brillouin measurements.^30^ This dependence on corneal thickness and hydration introduces challenges because unknown variability in hydration in a general patient population can confound Brillouin measurements.

Overall, our results suggest that PS-OCT, through PR and ultrahigh-resolution sublayer imaging, can improve early diagnosis of KC and may allow certain SKC eyes to be reclassified as healthy, increasing eligibility for refractive procedures. However, these conclusions need to be confirmed by prospective long-term studies across larger and more diverse populations. With further refinement and wider adoption, PS-OCT could provide more clinically meaningful diagnostic tools beyond conventional tomography, potentially reshaping the evaluation and management of keratoconus.

## 5 Conclusion

This study demonstrates that ultrahigh-resolution PS-OCT imaging may provide a clinically valuable, complementary approach to conventional tomography (Pentacam and MS-39) by capturing fundamentally different polarization-sensitive and sublayer structural features. These unique PS-OCT-derived insights may have strong potential to refine the diagnosis and staging of healthy, SKC, and KC eyes, advancing early detection and patient management.

## Supplementary Material

10.1117/1.BIOS.3.1.015004.s01

## Data Availability

The dataset used to train the three AI models supporting this study’s conclusions is available on GitHub (https://github.com/rahulprashantpatil/ps_ai_paperdata). Additional information may be obtained from the authors upon reasonable request.
