# Supplementary material for: Advancing subclinical keratoconus detection using polarization-sensitive optical coherence tomography and artificial intelligence
Source: Biophotonics Discov. 2026 Feb 9;3(1):015004. doi: 10.1117/1.BIOS.3.1.015004 (PMC13052500; doi:10.1117/1.BIOS.3.1.015004)
Supplement: Supplementary file 1 [file BIOS_003_015004_SD001.pdf]

Captions:

eFigure S1: Zernike polynomials pyramid

eTable S1(a): PS-OCT input features

(i) Phase Retardation
(ii) Epithelium layer
(iii) Bowman’s layer

eTable S1(b): Pentacam input features

eTable S1(c): MS-39 input features.

eTable S2(a): Classification of individual cases of the fellow eye of highly asymmetric (FHA) keratoconus subjects.

eTable S2(b): Classification of individual cases of the bilateral suspect keratoconus subjects.

eFigure 1: Zernike polynomials pyramid

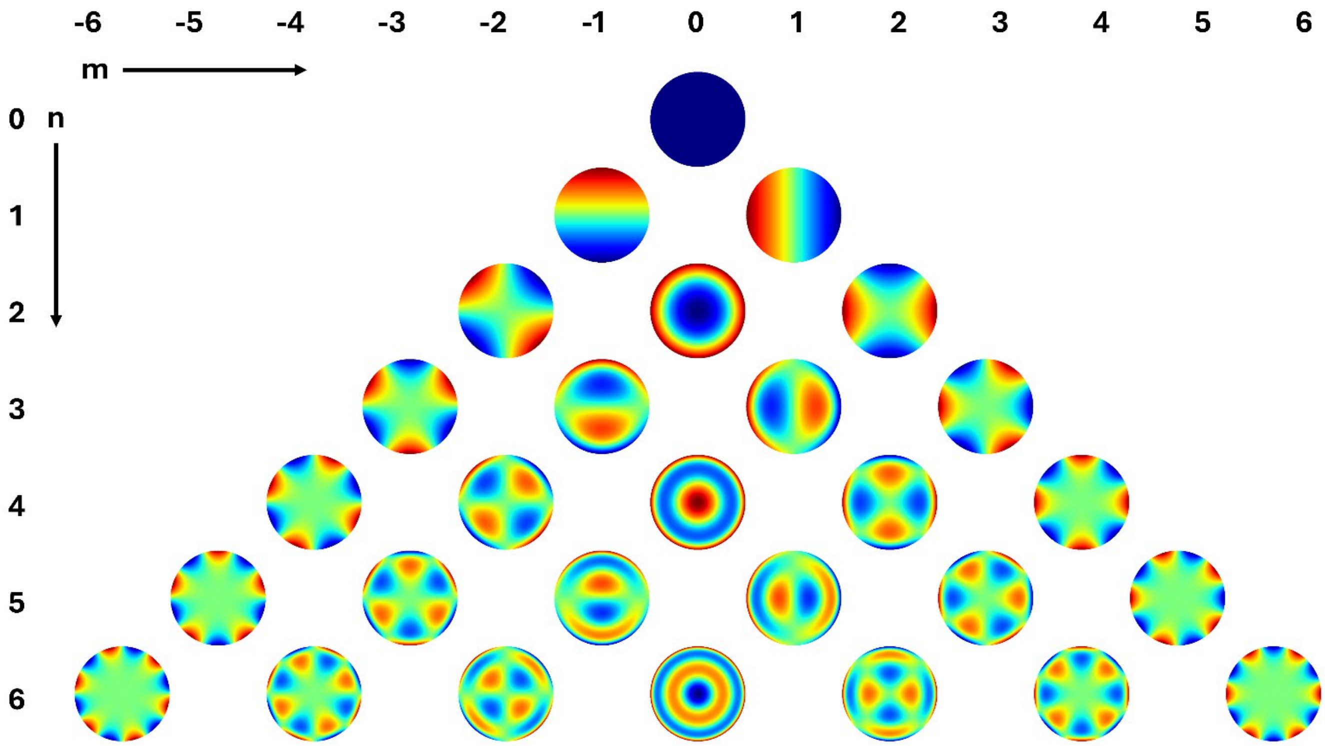

The Zernike polynomials are represented as Z_n^m. A double indexing scheme is useful for unambiguously describing the functions, with the index n describing the order of the radial polynomial and the index m describing the azimuthal frequency of the azimuthal component.

Table S1(a): PS-OCT input features

(i) Phase retardation.

N	Spatial Features (n = 28)	Healthy (n=120)	Sub-clinical (n=109)	Keratoconus (n=130)	p-value*
1	Z_0^0	40.05 ± 3.35 (31.75 to 47.90)	42.56 ± 4.38 (30.98 to 52.52)	42.86 ± 3.70 (33.09 to 51.25)	<0.001[a]
2	Z_1^1	0.32 ± 2.30 (-4.71 to 5.71)	0.39 ± 2.17 (-6.93 to 4.75)	0.23 ± 2.54 (-7.21 to 4.97)	0.878
3	Z_1^{-1}	-2.18 ± 2.00 (-7.77 to 3.60)	-2.39 ± 1.94 (-6.76 to 1.80)	-1.17 ± 2.78 (-9.45 to 5.41)	<0.001[b]
4	Z_2^2	-1.29 ± 1.73 (-5.34 to 3.12)	-1.47 ± 2.30 (-7.03 to 3.71)	-2.93 ± 2.81 (-17.90 to 3.02)	<0.001[b]
5	Z_2^0	7.47 ± 1.54 (3.35 to 11.29)	8.09 ± 2.01 (3.23 to 12.65)	8.02 ± 1.92 (3.42 to 15.46)	0.019[c]
6	Z_2^{-2}	0.23 ± 2.38 (-6.59 to 6.67)	0.34 ± 2.11 (-5.38 to 5.69)	0.41 ± 2.73 (-7.17 to 7.96)	0.834
7	Z_3^3	-0.04 ± 1.18 (-2.56 to 3.50)	0.07 ± 1.14 (-3.07 to 2.87)	0.09 ± 1.42 (-3.25 to 3.24)	0.665
8	Z_1^3	0.02 ± 1.26 (-2.06 to 3.40)	0.29 ± 1.54 (-2.87 to 4.61)	0.34 ± 1.48 (-3.22 to 4.14)	0.173
9	Z_{-1}^3	0.99 ± 1.30 (-1.97 to 4.70)	1.07 ± 1.50 (-3.44 to 4.57)	-0.54 ± 1.48 (-5.24 to 3.76)	<0.001[b]
10	Z_{-3}^3	0.04 ± 1.24 (-3.61 to 2.50)	0.27 ± 1.70 (-3.38 to 6.40)	0.71 ± 1.59 (-3.28 to 8.61)	0.002[d]
11	Z_4^4	-1.85 ± 1.26 (-5.50 to 0.63)	-0.92 ± 1.88 (-6.07 to 3.48)	-0.86 ± 1.82 (-6.58 to 7.10)	<0.001[a]
12	Z_2^4	0.94 ± 1.02 (-1.46 to 4.51)	0.63 ± 1.35 (-2.47 to 3.79)	0.43 ± 1.50 (-7.57 to 4.42)	0.010[d]
13	Z_0^4	-2.30 ± 0.98 (-5.54 to 0.05)	-2.59 ± 1.15 (-5.18 to 2.06)	-1.71 ± 1.50 (-5.64 to 5.72)	<0.001[b]
14	Z_{-2}^4	0.19 ± 1.42 (-2.85 to 3.70)	-0.02 ± 1.52 (-3.17 to 3.44)	0.04 ± 1.80 (-3.94 to 4.34)	0.599
15	Z_{-4}^4	-0.05 ± 0.81 (-1.96 to 2.28)	-0.18 ± 0.75 (-1.75 to 1.69)	-0.21 ± 0.97 (-2.08 to 2.79)	0.288
16	Z_5^5	-0.07 ± 0.79 (-1.94 to 3.00)	0.05 ± 0.87 (-2.23 to 2.81)	0.10 ± 0.84 (-2.82 to 2.02)	0.259
17	Z_3^5	0.27 ± 0.89 (-2.23 to 2.07)	0.23 ± 0.95 (-2.82 to 2.89)	0.22 ± 1.16 (-1.91 to 2.46)	0.926
18	Z_1^5	-0.03 ± 0.41 (-1.09 to 1.42)	-0.31 ± 0.69 (-3.63 to 1.11)	-0.23 ± 0.58 (-2.35 to 1.36)	0.001[a]
19	Z_{-1}^5	-0.18 ± 0.76 (-2.15 to 1.35)	-0.68 ± 1.32 (-7.06 to 1.42)	-0.64 ± 1.06 (-4.36 to 1.98)	<0.001[a]
20	Z_{-3}^5	0.54 ± 0.79 (-1.55 to 2.26)	0.66 ± 0.83 (-1.00 to 3.25)	0.83 ± 1.15 (-2.12 to 4.66)	0.047[d]
21	Z_{-5}^5	-1.02 ± 1.00 (-4.26 to 1.68)	-1.24 ± 1.21 (-5.14 to 1.21)	-1.12 ± 1.39 (-6.40 to 1.45)	0.404
22	Z_6^6	-0.36 ± 0.63 (-1.94 to 1.22)	-0.68 ± 0.87 (-3.09 to 1.18)	-1.00 ± 1.07 (-7.01 to 1.06)	<0.001[e]
23	Z_4^6	0.92 ± 0.69 (-0.67 to 3.24)	1.25 ± 0.91 (-0.45 to 3.90)	1.58 ± 1.08 (-1.27 to 5.41)	<0.001[e]

24	Z_2^6	0.05 ± 0.55 (-1.36 to 1.53)	-0.42 ± 0.80 (-3.75 to 1.54)	-0.71 ± 1.03 (-5.12 to 1.46)	<0.001[e]
25	Z_0^6	0.51 ± 0.59 (-0.84 to 1.99)	0.76 ± 0.77 (-0.94 to 2.85)	0.91 ± 0.80 (-1.48 to 3.45)	<0.001[a]
26	Z_{-2}^6	-0.08 ± 0.46 (-2.14 to 1.30)	-0.01 ± 0.95 (-4.24 to 3.55)	-0.03 ± 0.82 (-3.50 to 3.08)	0.756
27	Z_{-4}^6	-0.01 ± 0.59 (-1.64 to 2.19)	-0.01 ± 0.76 (-3.26 to 3.92)	-0.01 ± 0.69 (-2.15 to 2.25)	0.998
28	Z_{-6}^6	0.27 ± 0.77 (-2.55 to 2.01)	0.34 ± 0.96 (-3.25 to 3.65)	0.29 ± 1.15 (-2.72 to 4.30)	0.831

[*] One-Way ANOVA

[a]Healthy vs Sub-clinical KC and Keratoconus; [b]Keratoconus vs Healthy and Sub-clinical KC; [c]Healthy vs Sub-clinical KC; [d]Healthy vs Keratoconus; [e]Healthy vs Sub-clinical KC vs Keratoconus

The Zernike polynomials are represented as Z_n^m. A double indexing scheme is useful for unambiguously describing the functions, with the index n describing the order of the radial polynomial and the index m describing the azimuthal frequency of the azimuthal component.

Data is represented as Mean ± Standard Deviation, and range as (min to max) appear in parentheses.

(ii) Epithelium layer.

N	Spatial Features (n = 28)	Healthy (n=120)	Sub-clinical (n=109)	Keratoconus (n=130)	p-value*
1	Z_0^0	53.00 ± 2.78 (46.53 to 59.84)	51.97 ± 3.24 (44.19 to 60.04)	52.59 ± 3.07 (44.55 to 59.18)	0.038[a]
2	Z_1^1	0.02 ± 0.58 (-1.82 to 1.31)	0.00 ± 0.75 (-2.08 to 2.31)	-0.06 ± 0.70 (-1.87 to 2.37)	0.620
3	Z_1^{-1}	1.04 ± 1.11 (-1.94 to 4.69)	1.17 ± 1.10 (-1.50 to 4.39)	0.47 ± 1.23 (-2.39 to 4.70)	<0.001[b]
4	Z_2^2	0.48 ± 0.77 (-1.35 to 3.24)	0.49 ± 0.76 (-1.40 to 3.36)	0.30 ± 0.78 (-2.11 to 2.63)	0.074
5	Z_2^0	-0.25 ± 0.92 (-3.06 to 2.53)	-0.20 ± 0.91 (-2.79 to 1.86)	0.90 ± 1.02 (-1.45 to 3.38)	<0.001[b]
6	Z_2^{-2}	-0.03 ± 0.36 (-1.11 to 1.10)	0.03 ± 0.36 (-1.22 to 1.42)	-0.05 ± 0.51 (-1.58 to 1.64)	0.309
7	Z_3^3	0.03 ± 0.25 (-0.58 to 1.02)	0.04 ± 0.27 (-0.58 to 0.90)	0.02 ± 0.32 (-1.29 to 0.73)	0.815
8	Z_1^3	-0.01 ± 0.16 (-0.48 to 0.35)	0.01 ± 0.21 (-0.57 to 0.86)	-0.04 ± 0.70 (-1.99 to 2.18)	0.630
9	Z_{-1}^3	0.10 ± 0.36 (-1.09 to 1.17)	0.23 ± 0.43 (-0.98 to 1.91)	1.09 ± 0.71 (-0.54 to 3.14)	<0.001[b]
10	Z_{-3}^3	-0.19 ± 0.32 (-1.12 to 0.95)	-0.31 ± 0.41 (-2.04 to 0.57)	-0.29 ± 0.46 (-1.42 to 0.96)	0.042[a]
11	Z_4^4	0.03 ± 0.24 (-0.57 to 0.88)	0.01 ± 0.29 (-0.86 to 0.77)	0.11 ± 0.38 (-0.87 to 0.99)	0.017[c]
12	Z_2^4	0.12 ± 0.24 (-0.51 to 0.68)	0.16 ± 0.30 (-0.66 to 1.37)	-0.15 ± 0.51 (-1.44 to 1.30)	<0.001[b]
13	Z_0^4	-0.09 ± 0.30 (-0.78 to 0.73)	-0.07 ± 0.39 (-1.42 to 0.89)	-0.82 ± 0.86 (-3.67 to 0.74)	<0.001[b]
14	Z_{-2}^4	0.03 ± 0.13 (-0.29 to 0.36)	0.02 ± 0.16 (-0.35 to 0.56)	0.08 ± 0.57 (-1.32 to 2.19)	0.433
15	Z_{-4}^4	-0.01 ± 0.15 (-0.35 to 0.41)	-0.03 ± 0.22 (-0.78 to 0.76)	-0.03 ± 0.25 (-0.61 to 0.83)	0.704
16	Z_5^5	-0.04 ± 0.11 (-0.36 to 0.29)	-0.02 ± 0.18 (-0.90 to 0.36)	0.01 ± 0.19 (-0.50 to 0.61)	0.075
17	Z_3^5	0.01 ± 0.12 (-0.30 to 0.46)	0.00 ± 0.14 (-0.35 to 0.56)	-0.03 ± 0.29 (-0.79 to 0.69)	0.291
18	Z_1^5	-0.01 ± 0.10 (-0.24 to 0.30)	0.00 ± 0.10 (-0.22 to 0.31)	0.01 ± 0.55 (-1.89 to 1.61)	0.938
19	Z_{-1}^5	-0.06 ± 0.20 (-0.50 to 0.43)	-0.11 ± 0.26 (-1.12 to 0.46)	-1.14 ± 0.73 (-3.45 to 1.10)	<0.001[b]
20	Z_{-3}^5	0.10 ± 0.16 (-0.36 to 0.60)	0.14 ± 0.19 (-0.37 to 0.77)	0.40 ± 0.42 (-0.91 to 1.47)	<0.001[b]
21	Z_{-5}^5	-0.10 ± 0.16 (-0.47 to 0.49)	-0.11 ± 0.21 (-0.74 to 0.52)	-0.22 ± 0.26 (-0.95 to 0.54)	<0.001[b]
22	Z_6^6	-0.08 ± 0.09 (-0.35 to 0.17)	-0.09 ± 0.14 (-0.50 to 0.25)	-0.09 ± 0.16 (-0.59 to 0.36)	0.810
23	Z_4^6	0.06 ± 0.10 (-0.30 to 0.40)	0.06 ± 0.12 (-0.27 to 0.36)	0.09 ± 0.20 (-0.33 to 0.65)	0.124
24	Z_2^6	-0.09 ± 0.11 (-0.51 to 0.14)	-0.09 ± 0.15 (-0.60 to 0.27)	-0.03 ± 0.42 (-1.21 to 1.08)	0.158

25	Z_0^6	0.17 ± 0.11 (-0.04 to 0.47)	0.17 ± 0.16 (-0.21 to 1.09)	0.57 ± 0.59 (-0.71 to 2.26)	<0.001[b]
26	Z^6_{-2}	0.00 ± 0.08 (-0.20 to 0.29)	-0.00 ± 0.12 (-0.24 to 0.66)	-0.05 ± 0.42 (-1.55 to 0.87)	0.196
27	Z^6_{-4}	-0.02 ± 0.08 (-0.33 to 0.15)	-0.00 ± 0.09 (-0.31 to 0.18)	0.02 ± 0.17 (-0.43 to 0.43)	0.090
28	Z^6_{-6}	0.02 ± 0.10 (-0.29 to 0.37)	0.01 ± 0.13 (-0.35 to 0.40)	-0.01 ± 0.13 (-0.47 to 0.27)	0.139

[*] One-Way ANOVA

[a]Healthy vs Sub-clinical KC; [b]Keratoconus vs Healthy and Sub-clinical KC; [c]Sub-clinical KC vs Keratoconus

The Zernike polynomials are represented as Z_n^m. A double indexing scheme is useful for unambiguously describing the functions, with the index n describing the order of the radial polynomial and the index m describing the azimuthal frequency of the azimuthal component.

Data is represented as Mean ± Standard Deviation, and range as (min to max) appear in parentheses.

(iii) Bowman's layer.

N	Spatial Features (n = 28)	Healthy (n=120)	Sub-clinical (n=109)	Keratoconus (n=130)	p-value*
1	Z_0^0	15.56 ± 1.28 (12.87 to 19.07)	15.58 ± 1.52 (12.72 to 20.10)	15.01 ± 1.37 (12.15 to 19.44)	0.001[a]
2	Z_1^1	0.01 ± 0.17 (-0.34 to 0.43)	-0.02 ± 0.17 (-0.45 to 0.43)	0.02 ± 0.23 (-0.49 to 0.98)	0.221
3	Z_1^{-1}	-0.04 ± 0.22 (-0.65 to 0.60)	-0.06 ± 0.24 (-0.47 to 0.73)	-0.49 ± 0.43 (-2.06 to 0.55)	<0.001[a]
4	Z_2^2	-0.01 ± 0.15 (-0.35 to 0.54)	0.02 ± 0.23 (-0.64 to 0.70)	0.05 ± 0.23 (-0.52 to 0.90)	0.113
5	Z_2^0	-0.27 ± 0.31 (-0.96 to 0.41)	-0.28 ± 0.37 (-1.69 to 0.38)	0.15 ± 0.42 (-0.81 to 1.33)	<0.001[a]
6	Z_2^{-2}	-0.02 ± 0.10 (-0.42 to 0.22)	-0.01 ± 0.12 (-0.30 to 0.37)	-0.00 ± 0.15 (-0.43 to 0.48)	0.417
7	Z_3^3	-0.02 ± 0.08 (-0.23 to 0.18)	-0.01 ± 0.10 (-0.29 to 0.36)	-0.01 ± 0.11 (-0.42 to 0.31)	0.961
8	Z_1^3	-0.03 ± 0.07 (-0.19 to 0.18)	-0.01 ± 0.09 (-0.18 to 0.25)	-0.02 ± 0.14 (-0.57 to 0.32)	0.574
9	Z_{-1}^3	0.05 ± 0.12 (-0.23 to 0.42)	0.04 ± 0.15 (-0.61 to 0.49)	0.31 ± 0.24 (-0.28 to 0.99)	<0.001[a]
10	Z_{-3}^3	-0.05 ± 0.12 (-0.40 to 0.23)	-0.04 ± 0.17 (-0.58 to 0.65)	-0.06 ± 0.17 (-0.97 to 0.31)	0.607
11	Z_4^4	-0.02 ± 0.10 (-0.27 to 0.21)	-0.03 ± 0.13 (-0.49 to 0.67)	-0.01 ± 0.11 (-0.54 to 0.38)	0.296
12	Z_2^4	0.02 ± 0.08 (-0.19 to 0.29)	0.02 ± 0.12 (-0.56 to 0.34)	-0.04 ± 0.13 (-0.34 to 0.54)	<0.001[a]
13	Z_0^4	-0.02 ± 0.12 (-0.33 to 0.21)	0.03 ± 0.13 (-0.37 to 0.58)	-0.12 ± 0.18 (-0.70 to 0.33)	<0.001[b]
14	Z_{-2}^4	0.00 ± 0.05 (-0.16 to 0.15)	-0.01 ± 0.07 (-0.30 to 0.17)	0.01 ± 0.11 (-0.26 to 0.37)	0.136
15	Z_{-4}^4	0.01 ± 0.09 (-0.20 to 0.23)	0.02 ± 0.09 (-0.24 to 0.25)	0.02 ± 0.08 (-0.23 to 0.27)	0.266
16	Z_5^5	-0.00 ± 0.07 (-0.18 to 0.21)	0.00 ± 0.09 (-0.16 to 0.37)	0.01 ± 0.08 (-0.22 to 0.25)	0.639
17	Z_3^5	0.01 ± 0.05 (-0.20 to 0.15)	-0.01 ± 0.06 (-0.28 to 0.17)	-0.01 ± 0.07 (-0.18 to 0.22)	0.095
18	Z_1^5	-0.01 ± 0.04 (-0.12 to 0.10)	-0.01 ± 0.05 (-0.14 to 0.15)	-0.00 ± 0.07 (-0.19 to 0.17)	0.460
19	Z_{-1}^5	0.00 ± 0.07 (-0.20 to 0.28)	-0.02 ± 0.10 (-0.49 to 0.24)	-0.11 ± 0.14 (-0.52 to 0.25)	<0.001[a]
20	Z_{-3}^5	-0.01 ± 0.07 (-0.26 to 0.16)	-0.01 ± 0.08 (-0.21 to 0.27)	0.02 ± 0.08 (-0.30 to 0.22)	0.007[a]
21	Z_{-5}^5	0.02 ± 0.08 (-0.16 to 0.29)	0.02 ± 0.09 (-0.31 to 0.33)	0.01 ± 0.09 (-0.21 to 0.42)	0.383
22	Z_6^6	-0.00 ± 0.06 (-0.20 to 0.29)	0.01 ± 0.07 (-0.18 to 0.26)	0.01 ± 0.08 (-0.20 to 0.24)	0.233
23	Z_4^6	-0.01 ± 0.05 (-0.21 to 0.13)	0.00 ± 0.05 (-0.13 to 0.18)	-0.02 ± 0.07 (-0.22 to 0.20)	0.05[c]
24	Z_2^6	0.01 ± 0.05 (-0.12 to 0.16)	-0.00 ± 0.06 (-0.26 to 0.22)	0.04 ± 0.08 (-0.32 to 0.24)	<0.001[a]

25	Z_0^6	0.03 ± 0.06 (-0.15 to 0.19)	0.05 ± 0.08 (-0.14 to 0.40)	0.01 ± 0.09 (-0.36 to 0.31)	0.003[c]
26	Z^6_{-2}	-0.00 ± 0.04 (-0.10 to 0.08)	-0.00 ± 0.05 (-0.17 to 0.14)	-0.01 ± 0.06 (-0.34 to 0.12)	0.291
27	Z^6_{-4}	0.00 ± 0.05 (-0.15 to 0.13)	0.01 ± 0.06 (-0.12 to 0.28)	0.00 ± 0.05 (-0.13 to 0.14)	0.651
28	Z^6_{-6}	0.00 ± 0.06 (-0.20 to 0.22)	0.01 ± 0.08 (-0.30 to 0.19)	0.00 ± 0.06 (-0.15 to 0.20)	0.850

[*] One-Way ANOVA

[a]Keratoconus vs Healthy and Sub-clinical KC; [b]Healthy vs Sub-clinical KC vs Keratoconus; [c]Sub-clinical KC vs Keratoconus.

The Zernike polynomials are represented as Z_n^m. A double indexing scheme is useful for unambiguously describing the functions, with the index n describing the order of the radial polynomial and the index m describing the azimuthal frequency of the azimuthal component.

Data is represented as Mean ± Standard Deviation, and range as (min to max) appear in parentheses.

Table S1(b): Pentacam input features

Features (n = 24)	Healthy (n=120)	Sub-clinical (n=109)	Keratoconus (n=130)	p-value*
**Keratometry**				
K1 (D)	42.63 ± 1.23 (40.20 to 46.70)	44.07 ± 1.65 (40.30 to 48.20)	45.61 ± 3.13 (38.50 to 58.80)	<0.001[a]
K2 (D)	43.98 ± 1.20 (41.70 to 47.30)	45.44 ± 1.86 (41.10 to 49.40)	49.18 ± 3.97 (40.80 to 65.00)	<0.001[a]
K mean (D)	43.28 ± 1.12 (41.30 to 47.00)	44.74 ± 1.70 (40.70 to 48.70)	47.31 ± 3.42 (40.00 to 61.80)	<0.001[a]
K max (D)	44.46 ± 1.22 (42.10 to 47.80)	46.01 ± 1.94 (41.40 to 50.30)	54.96 ± 4.86 (45.50 to 72.00)	<0.001[a]
Axis (flat)	91.03 ± 76.83 (0.40 to 179.40)	94.71 ± 79.09 (0.20 to 179.70)	98.77 ± 64.74 (0.20 to 179.80)	0.707
Astigmatism (D)	1.35 ± 0.93 (0.10 to 4.30)	1.31 ± 0.90 (-1.10 to 4.20)	3.56 ± 1.87 (0.10 to 9.60)	<0.001[b]
**Tomographic parameters (Pentacam derived indices)**				
ISV	17.91 ± 5.90 (7.00 to 38.00)	20.44 ± 6.10 (9.00 to 39.00)	80.64 ± 26.08 (39.00 to 175.00)	<0.001[b]
IVA	0.11 ± 0.04 (0.03 to 0.23)	0.13 ± 0.06 (0.02 to 0.35)	0.88 ± 0.33 (0.43 to 2.01)	<0.001[b]
KI	1.02 ± 0.02 (0.98 to 1.07)	1.03 ± 0.02 (0.98 to 1.07)	1.22 ± 0.09 (1.09 to 1.61)	<0.001[b]
CKI	1.01 ± 0.01 (1.00 to 1.02)	1.01 ± 0.01 (0.99 to 1.02)	1.05 ± 0.04 (0.95 to 1.20)	<0.001[b]
IHA	5.98 ± 4.10 (0.00 to 18.80)	6.58 ± 5.12 (0.00 to 21.00)	36.02 ± 22.88 (0.00 to 95.60)	<0.001[b]
IHD	0.01 ± 0.01 (0.00 to 0.03)	0.01 ± 0.01 (0.00 to 0.03)	0.12 ± 0.05 (0.05 to 0.27)	<0.001[b]
IS Value	0.30 ± 0.48 (-0.99 to 1.33)	0.44 ± 0.49 (-0.68 to 1.36)	5.79 ± 2.33 (3.02 to 13.43)	<0.001[b]
KISA	8.38 ± 10.84 (0.33 to 53.33)	8.18 ± 10.34 (0.33 to 51.83)	911.67 ± 1288.58 (81.27 to 6663.67)	<0.001[b]
**Corneal aberrations**				
RMS HOA (µm)	0.36 ± 0.08 (0.22 to 0.58)	0.41 ± 0.11 (0.19 to 0.89)	2.70 ± 0.99 (1.27 to 5.96)	<0.001[b]
RMS LOA (µm)	1.81 ± 0.77 (0.74 to 4.56)	1.93 ± 0.67 (0.82 to 4.28)	10.42 ± 3.88 (4.17 to 21.65)	<0.001[b]

Astigmatism 0° (µm)	-1.12 ± 0.96 (-4.28 to 1.13)	-1.22 ± 0.85 (-3.80 to 0.75)	-2.37 ± 1.86 (-7.46 to 2.80)	<0.001[b]
Defocus (µm)	0.80 ± 0.32 (0.01 to 1.75)	0.83 ± 0.48 (-0.35 to 2.55)	-1.63 ± 3.38 (-13.54 to 5.71)	<0.001[b]
Astigmatism 45° (µm)	-0.02 ± 0.63 (-2.15 to 2.18)	-0.01 ± 0.55 (-1.83 to 1.34)	0.25 ± 2.08 (-4.89 to 4.97)	0.197
Trefoil 0° (µm)	0.01 ± 0.10 (-0.29 to 0.24)	-0.00 ± 0.09 (-0.30 to 0.36)	0.07 ± 0.41 (-1.21 to 1.58)	0.05
Coma 0° (µm)	-0.00 ± 0.13 (-0.29 to 0.39)	-0.01 ± 0.19 (-0.52 to 0.46)	-0.08 ± 0.92 (-3.16 to 2.80)	0.450
Coma 90° (µm)	-0.09 ± 0.15 (-0.46 to 0.37)	-0.12 ± 0.17 (-0.45 to 0.35)	-2.23 ± 0.89 (-5.16 to -1.04)	<0.001[b]
Trefoil 30° (µm)	-0.04 ± 0.09 (-0.26 to 0.13)	-0.03 ± 0.10 (-0.26 to 0.35)	0.32 ± 0.37 (-0.66 to 1.33)	<0.001[b]
Spherical aberration (µm)	0.22 ± 0.07 (0.07 to 0.38)	0.22 ± 0.11 (-0.04 to 0.60)	-0.41 ± 0.69 (-2.47 to 1.15)	<0.001[b]

[*] One-Way ANOVA

[a]KC vs Healthy vs Sub-clinical KC; [b]KC vs Healthy and Sub-clinical KC

K1- flat keratometry; K2- Steep keratometry; K mean- mean keratometry; K max- maximum keratometry; ISV- Index of surface variance; IVA- Index of vertical asymmetry; KI- Keratoconus index; CKI- Centre keratoconus index; IHA- Index of height asymmetry; IHD- Index of height decentration; IS value- Difference between inferior and superior corneal power (3 mm zone).

Aberrations were evaluated with Zernike polynomials up to 6th order (central 6 mm cornea); RMS LOA- Root mean square of lower order aberrations; RMS HOA- root mean square of higher order aberrations.

Data is represented as Mean ± Standard Deviation, and range as (min to max) appear in parentheses.

Table S1(c): MS-39 input features.

Features (n = 23)	**Healthy (n=120)**	**Sub-clinical (n=109)**	**Keratoconus (n=130)**	**p-value***
**Anterior surface keratometry and wavefront aberrations**				
AntK1 (D)	44.58 ± 1.19 (42.06 to 47.72)	46.13 ± 1.87 (41.61 to 49.60)	53.67 ± 4.57 (43.38 to 74.66)	<0.001[a]
AntK2 (D)	43.08 ± 1.23 (40.33 to 46.78)	44.56 ± 1.66 (40.96 to 47.80)	46.47 ± 3.61 (38.61 to 63.47)	<0.001[a]
AntAxis	94.71 ± 23.60 (39.37 to 175.78)	90.21 ± 23.35 (11.25 to 174.38)	89.06 ± 21.25 (2.81 to 180.00)	0.122
AntKmax (D)	44.75 ± 1.21 (42.26 to 48.02)	46.35 ± 1.93 (41.83 to 50.22)	54.58 ± 4.65 (45.53 to 75.04)	<0.001[a]
AntLORMS (µm)	1.41 ± 0.48 (0.52 to 4.02)	1.44 ± 0.53 (0.33 to 2.81)	5.26 ± 2.91 (0.99 to 14.89)	<0.001[b]
AntHORMS (µm)	0.53 ± 0.14 (0.30 to 1.50)	0.61 ± 0.14 (0.34 to 1.10)	3.18 ± 1.23 (1.44 to 8.72)	<0.001[b]
AntComaRMS (µm)	0.26 ± 0.11 (0.02 to 0.55)	0.34 ± 0.16 (0.02 to 0.81)	2.73 ± 1.19 (1.22 to 7.90)	<0.001[b]
AntDefocus (µm)	1.32 ± 0.44 (-0.01 to 2.74)	1.32 ± 0.57 (-0.20 to 2.78)	-1.36 ± 4.13 (-13.66 to 7.16)	<0.001[b]
AntSA (µm)	0.32 ± 0.10 (0.05 to 0.57)	0.33 ± 0.16 (-0.01 to 0.76)	-0.30 ± 0.76 (-3.07 to 1.35)	<0.001[b]
**Epithelium-Bowman Interface (BW) keratometry and wavefront aberrations**				
BWK1 (D)	44.87 ± 1.22 (42.46 to 48.20)	46.47 ± 1.86 (42.00 to 50.32)	56.02 ± 4.68 (42.93 to 71.04)	<0.001[a]
BWK2 (D)	43.32 ± 1.33 (39.98 to 46.94)	44.72 ± 1.65 (41.03 to 48.26)	47.28 ± 4.33 (37.26 to 60.85)	<0.001[a]
BWAxis	91.75 ± 24.74 (0.00 to 180.00)	91.03 ± 20.77 (18.28 to 160.31)	90.10 ± 19.79 (5.63 to 175.78)	0.836
BWKmax (D)	45.31 ± 1.32 (42.59 to 48.63)	46.97 ± 1.87 (42.01 to 51.27)	57.23 ± 4.81 (47.17 to 71.14)	<0.001[a]
BWLORMS (µm)	1.38 ± 0.86 (0.13 to 4.92)	1.48 ± 0.83 (0.29 to 4.12)	7.67 ± 3.80 (1.15 to 22.45)	<0.001[b]
BWHORMS (µm)	0.52 ± 0.15 (0.24 to 1.53)	0.67 ± 0.19 (0.26 to 1.28)	4.04 ± 1.34 (2.22 to 8.44)	<0.001[b]
BWComaRMS (µm)	0.27 ± 0.12 (0.02 to 0.55)	0.41 ± 0.20 (0.02 to 1.06)	3.41 ± 1.32 (1.48 to 7.86)	<0.001[b]
BWDefocus (µm)	0.85 ± 1.24 (-1.77 to 4.42)	0.92 ± 1.20 (-1.68 to 4.07)	-2.75 ± 5.56 (-21.39 to 9.02)	<0.001[b]
BWSA (µm)	0.24 ± 0.15 (-0.18 to 0.71)	0.29 ± 0.22 (-0.18 to 0.78)	-0.55 ± 0.96 (-3.55 to 1.63)	<0.001[b]
**Epithelium Zernike indices (EZI)**				
eLORMS (µm)	1.23 ± 0.72 (0.22 to 3.29)	1.10 ± 0.65 (0.24 to 4.72)	2.01 ± 0.95 (0.44 to 5.35)	<0.001[b]
eHORMS (µm)	0.79 ± 0.25 (0.39 to 1.76)	0.90 ± 0.36 (0.37 to 2.52)	3.03 ± 1.00 (0.91 to 5.85)	<0.001[b]
eComaRMS (µm)	0.31 ± 0.19 (0.01 to 0.91)	0.41 ± 0.29 (0.03 to 1.58)	2.12 ± 0.90 (0.21 to 4.86)	<0.001[b]

eDefocus (μm)	-0.80 ± 1.03 (-3.13 to 1.98)	-0.74 ± 0.87 (-4.64 to 1.72)	0.45 ± 1.59 (-3.02 to 4.90)	<0.001[b]
eSA (μm)	-0.18 ± 0.39 (-1.14 to 0.84)	-0.10 ± 0.37 (-0.88 to 0.84)	-0.64 ± 0.97 (-4.67 to 1.89)	<0.001[b]

[*] One-Way ANOVA

[a]KC vs Healthy vs Sub-clinical KC; [b]KC vs Healthy and Sub-clinical KC

Order of the Zernike polynomials was 6 and the analyses diameter 6.4mm; K1 –steep axis curvature; K2 – flat axis curvature; LORMS – root mean square of lower order Zernike terms; HORMS – root mean square of higher order Zernike terms; SA – Primary spherical aberration (4th order); ComaRMS – root mean square of coma Zernike terms.

eTable S2(a): Classification of individual cases of the fellow eye of highly asymmetric (FHA) keratoconus subjects.

#	PS-OCT	Pentacam	MS-39
FHA#1	Healthy	SKC	SKC
FHA#2	Healthy	SKC	SKC
FHA#3	Healthy	SKC	SKC
FHA#4	Healthy	SKC	SKC
FHA#5	Healthy	Healthy	Healthy
FHA#6	Healthy	Healthy	Healthy
FHA#7	Healthy	Healthy	Healthy
FHA#8	SKC	SKC	SKC
FHA#9	SKC	Healthy	Healthy
FHA#10	Healthy	SKC	SKC
FHA#11	Healthy	SKC	SKC
FHA#12	SKC	SKC	SKC
FHA#13	SKC	Healthy	Healthy
FHA#14	SKC	SKC	SKC
FHA#15	SKC	SKC	Healthy
FHA#16	KC	SKC	SKC
FHA#17	SKC	SKC	SKC
FHA#18	Healthy	SKC	Healthy
FHA#19	SKC	SKC	SKC
FHA#20	Healthy	Healthy	Healthy
FHA#21	Healthy	Healthy	Healthy
FHA#22	Healthy	Healthy	Healthy
FHA#23	Healthy	SKC	SKC
FHA#24	SKC	SKC	SKC
FHA#25	SKC	Healthy	Healthy
FHA#26	Healthy	SKC	Healthy
FHA#27	Healthy	Healthy	Healthy
FHA#28	Healthy	SKC	Healthy
FHA#29	Healthy	Healthy	Healthy
FHA#30	Healthy	SKC	SKC
FHA#31	Healthy	Healthy	Healthy
FHA#32	Healthy	SKC	SKC
FHA#33	KC	SKC	SKC

eTable S2(b): Classification of individual cases of the bilateral suspect keratoconus subjects.

#	PS-OCT	Pentacam	MS-39
BI#1	SKC	SKC	SKC
BI#2	SKC	SKC	SKC
BI#3	SKC	SKC	SKC
BI#4	SKC	SKC	SKC
BI#5	SKC	SKC	SKC
BI#6	SKC	SKC	SKC
BI#7	SKC	Healthy	Healthy
BI#8	SKC	Healthy	SKC
BI#9	SKC	SKC	SKC
BI#10	SKC	SKC	SKC
BI#11	SKC	SKC	SKC
BI#12	SKC	SKC	SKC
BI#13	SKC	SKC	SKC
BI#14	Healthy	SKC	SKC
BI#15	Healthy	Healthy	Healthy
BI#16	Healthy	Healthy	Healthy
BI#17	Healthy	Healthy	Healthy
BI#18	Healthy	SKC	SKC
BI#19	Healthy	Healthy	Healthy
BI#20	Healthy	Healthy	Healthy
BI#21	SKC	SKC	SKC
BI#22	Healthy	SKC	Healthy
BI#23	SKC	SKC	SKC
BI#24	SKC	SKC	SKC
BI#25	SKC	SKC	SKC
BI#26	SKC	SKC	SKC
BI#27	SKC	Healthy	Healthy
BI#28	SKC	Healthy	Healthy
BI#29	SKC	SKC	SKC
BI#30	SKC	SKC	SKC
BI#31	Healthy	SKC	SKC
BI#32	SKC	SKC	SKC
BI#33	KC	Healthy	SKC
BI#34	SKC	Healthy	Healthy
BI#35	SKC	SKC	SKC
BI#36	SKC	SKC	SKC
BI#37	SKC	SKC	SKC
BI#38	SKC	SKC	SKC
BI#39	SKC	Healthy	Healthy
BI#40	SKC	Healthy	SKC
BI#41	SKC	SKC	SKC
BI#42	Healthy	SKC	SKC
BI#43	SKC	SKC	SKC
BI#44	SKC	SKC	SKC
BI#45	SKC	SKC	SKC

BI#46	SKC	SKC	SKC
BI#47	SKC	SKC	SKC
BI#48	SKC	SKC	SKC
BI#49	Healthy	SKC	Healthy
BI#50	Healthy	Healthy	SKC
BI#51	SKC	SKC	SKC
BI#52	SKC	SKC	SKC
BI#53	SKC	SKC	SKC
BI#54	SKC	SKC	SKC
BI#55	SKC	SKC	SKC
BI#56	SKC	SKC	SKC
BI#57	SKC	SKC	SKC
BI#58	SKC	SKC	SKC
BI#59	SKC	SKC	SKC
BI#60	SKC	SKC	SKC
BI#61	Healthy	SKC	SKC
BI#62	Healthy	SKC	SKC
BI#63	SKC	Healthy	Healthy
BI#64	SKC	Healthy	Healthy
BI#65	Healthy	SKC	SKC
BI#66	KC	SKC	Healthy
BI#67	SKC	SKC	SKC
BI#68	SKC	SKC	SKC
BI#69	Healthy	Healthy	Healthy
BI#70	Healthy	Healthy	Healthy
BI#71	Healthy	SKC	SKC
BI#72	SKC	SKC	SKC
BI#73	Healthy	SKC	Healthy
BI#74	Healthy	SKC	Healthy
BI#75	Healthy	SKC	SKC
BI#76	Healthy	SKC	SKC
